# Is Bisphosphonate-Related Osteonecrosis of the Jaw an Infection? A Histological and Microbiological Ten-Year Summary

**DOI:** 10.1155/2014/452737

**Published:** 2014-06-24

**Authors:** A. M. Hinson, C. W. Smith, E. R. Siegel, B. C. Stack

**Affiliations:** ^1^University of Arkansas for Medical Sciences (UAMS), College of Medicine, Little Rock, AR 72205, USA; ^2^Department of Otorhinolaryngology, The University of Oklahoma Health Sciences Center, Oklahoma City, OK 73104, USA; ^3^Department of Biostatistics, UAMS, Little Rock, AR 72205, USA; ^4^Department of Otolaryngology-Head and Neck Surgery, UAMS, 4301 W. Markham Street No. 543, Little Rock, AR 72205, USA

## Abstract

The role of infection in the etiology of bisphosphonate-related osteonecrosis of the jaw (BRONJ) is poorly understood. Large-scale epidemiological descriptions of the histology and microbiology of BRONJ are not found in the literature. Herein, we present a systematic review of BRONJ histology and microbiology (including demographics, immunocompromised associations, clinical signs and symptoms, disease severity, antibiotic and surgical treatments, and recovery status) validating that infection should still be considered a prime component in the multifactorial disease.

## 1. Introduction

In the early 20th century, phosphorus necrosis of the jaw or “phossy jaw” became clinically irrelevant after the manufacturing and importation of white phosphorous was banned in both Europe and the USA [1]. Then, in 2003, Marx described a previously unrecognized association between aminobisphosphonates (N-BPs) and ONJ rekindling interest in the seemingly familiar pathophysiology [2]. A thousand papers later, an association between N-BPs and ONJ is well documented. Yet, unlike white phosphorus, N-BPs are not so easily eliminated from human exposure. The benefits of N-BPs to patients suffering from severe osteoporosis, multiple myeloma, and/or metastatic tumors of the bone frequently outweigh the small but significant risk of ONJ. And, more recently, other drugs which are not bisphosphonates (e.g., denosumab) appear to share a similar presentation and pathophysiology, suggesting that the clinical relevance of ONJ is unlikely to diminish any time soon [3].

The mechanism of ONJ remains elusive at his time. Various hypotheses with convincing data suggest that inhibition of osteoclasts, diminished vascularity, direct tissue toxicity, impaired wound healing, microcracks, inflammation, and infection may all play at least some role in ONJ [4–9]. The latter is increasingly being recognized as a critical component in this multifactorial disease. However, controversy exists as to whether (1) N-BP inhibition of bone remodeling results in necrosis with subsequent infection or (2) the direct toxic effects of N-BPs on the oral mucosa allow for invasion of oral pathogens causing infection with subsequent necrosis [10].

Future advances with respect to the above debate will likely hinge on a finer appreciation for the unique setting in which BRONJ occurs. The oral cavity is perhaps the most susceptible of any anatomical location to the development of bone infection.

Repetitive mastication, tooth extraction, dental implantation, dentures, dental abscess, root canal surgery, and/or other oral trauma allow usually nonpathologic oral flora direct access to mandibular and maxillary bones [10]. In healthy individuals, a breech in the oral mucosa may be quickly overcome by proper wound healing leaving little possibility for continual infection. The patient population exposed to N-BPs, however, is typically immunocompromised in at least one of several ways including malignancy, chemotherapy, steroids, diabetes, and smoking.

A vast majority of the literature, however, has been limited to case reports/series with relatively little to no attention applied to histological and microbiological findings. The authors suggest that this is likely due to both (1) the difficulty in culturing several of the oral pathogens and (2) a previously held notion that BRONJ was mostly an aseptic process. Various modalities have been studied (i.e., imaging) to describe bone abnormalities seen with BRONJ but thus far have not proved reliable in describing the infectious nature of the disease [11]. Recent advances using biomolecular profiling to describe BRONJ flora (colonies of organisms typically invisible to standard techniques) have narrowed this gap [12]. Metagenomic analysis, while informative, has unfortunately been limited by relatively few numbers of analytical samples making interpretation of larger microbiological patterns associated with ONJ extremely difficult.

Herein, we present a summary of the current microbiological and histological data (including relevant demographic data) of all BRONJ cases reported in the literature in an attempt to describe the role microorganisms play in the pathophysiology of ONJ.

## 2. Materials and Methods

A protocol that specified the inclusion criteria used in the present systematic review was developed in advance and a review exemption from the UAMS IRB was obtained.

### 2.1. Selection Criteria and Search Strategy

Review articles that compiled data from multiple previously published sources were excluded. Case reports, case series, and/or case-control studies relevant to ONJ (written in English) from January 2003 to December 2013 were reviewed for histological and/or microbiological data. The PubMed/MEDLINE electronic database was searched (with an English language limitation) for any published case report, case series, and/or case-control studies. Various arrangements of “osteonecrosis” in conjunction with free text (*jaw*,* jawbone*,* mandible*,* maxilla*,* bisphosphonate*,* zoledronic acid*,* zoledronate*,* pamidronate*,* alendronate*,* ibandronate*,* risedronate*,* osteomyelitis*,* infection*,* histology*,* microbiology*,* cultures*,* molecular*,* metagenomic*, and* bioprofiling*) were entered into the search engine. Resulting titles and abstracts were then scanned for potentially eligible studies. The remaining articles were read in full to determine inclusion status.

### 2.2. Data Extraction and Analysis

Data was entered into Microsoft Excel according to the following categories (in brevity): (1) general: article name, number of cases; (2) demographics and history: gender, age, history of dental trigger, N-BP route, and BRONJ stage; (3) clinical manifestations: bone exposure, pain, erythema, pus, and other extraoral manifestations (lymphadenopathy, swelling, sinus tract, etc.); (4) treatment: antibiotic route/duration, surgical type; (5) outcome: recovery status, time to recovery; (6) relevant comorbidities: chemotherapy, steroids, diabetes, smoking, and neoplastic disease; (7) microscopic identification of* Actinomyces* via hematoxylin-eosin (H&E), Gram, and/or periodic acid-Schiff (PAS); (8) histological descriptions: specimen number, presence of necrotic bone, bacterial colonization, inflammatory infiltrate, osteolysis, and irregular/scalloped borders; (9) culture results: growth, no growth, and name of isolated phylotype. Each numbered variable listed above was defined by reported, nonreported, sum, mean, standard deviation, median, lower quartile, upper quartile, minimum, and maximum. Summary statistics were then analyzed.

## 3. Results

### 3.1. Search and Study Inclusion

**Figure 1:**
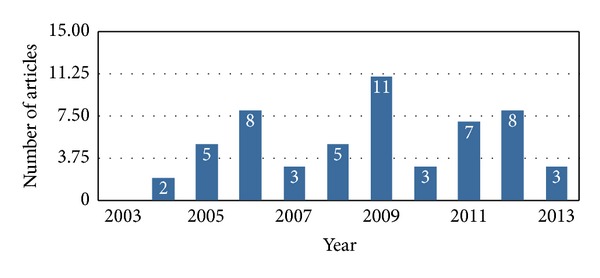
Published articles (*n* = 55) containing histological and microbiological data from January 2003 to December 2013.

**Figure 2:**
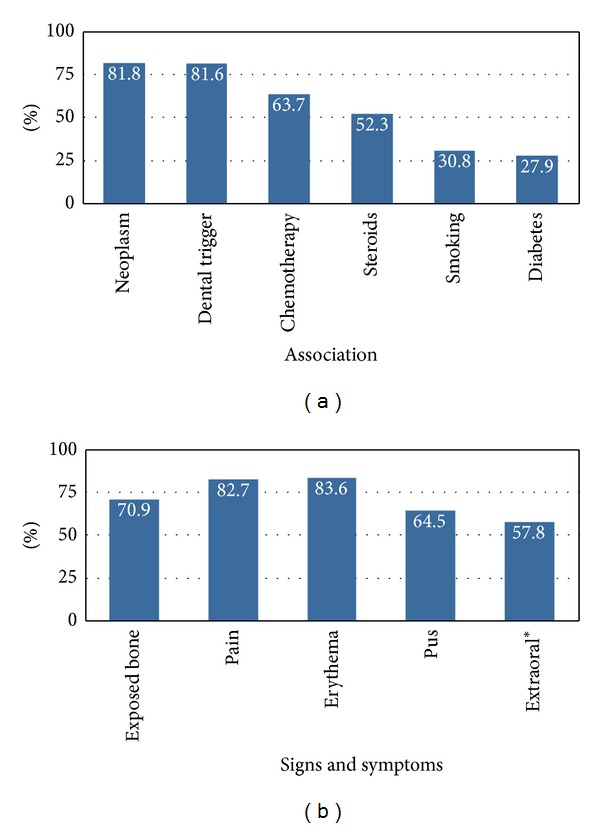
Immunosuppressed association (a) and clinical presentation (b) of the BRONJ population (*n* = 814).

More than 1,000 articles were initially identified by the PubMed/MEDLINE search. After exclusion of non-English articles, animal studies, and review articles, 175 papers were considered eligible and full-texts were carefully read. Articles without histological or microbiological data were excluded and this resulted in 55 articles including 814 patients. The number of eligible publications per year showed a bell-shaped distribution with a peak number of reports in 2009 (Figure 1). Age ranged from 26 to 89 years $\left(\overset{-}{x}=63.3+5.6\right)$ with a male to female ratio of 1 : 1.7 (264 males, 445 females). 95 (18.0%) and 516 (81.8%) had a history of oral or parenteral N-BP exposure, respectively. Previously reported risk factors/associations included 392 (81.8%) with neoplastic disease, 488 (81.6%) with recent history of dentoalveolar procedure, 245 (63.7%) treated with chemotherapy, 133 (52.3%) with steroid exposure, 32 (30.8%) tobacco users, and 38 (27.9%) with diabetes mellitus (Figure 2(a)). Extent of disease (BRONJ Stages I–III) was recorded in 210 cases with 25.7% (I), 57.6% (II), and 16.7% (III). Clinical manifestations included pain (82.7%, *n* = 321), bone exposure in (70.9%, *n* = 270), erythema (83.6%, *n* = 31), pus (64.5%, *n* = 109), and other extraoral manifestations in 101 (57.8%) (Figure 2(b)).

### 3.2. Histology and Microbiology

**Figure 3:**
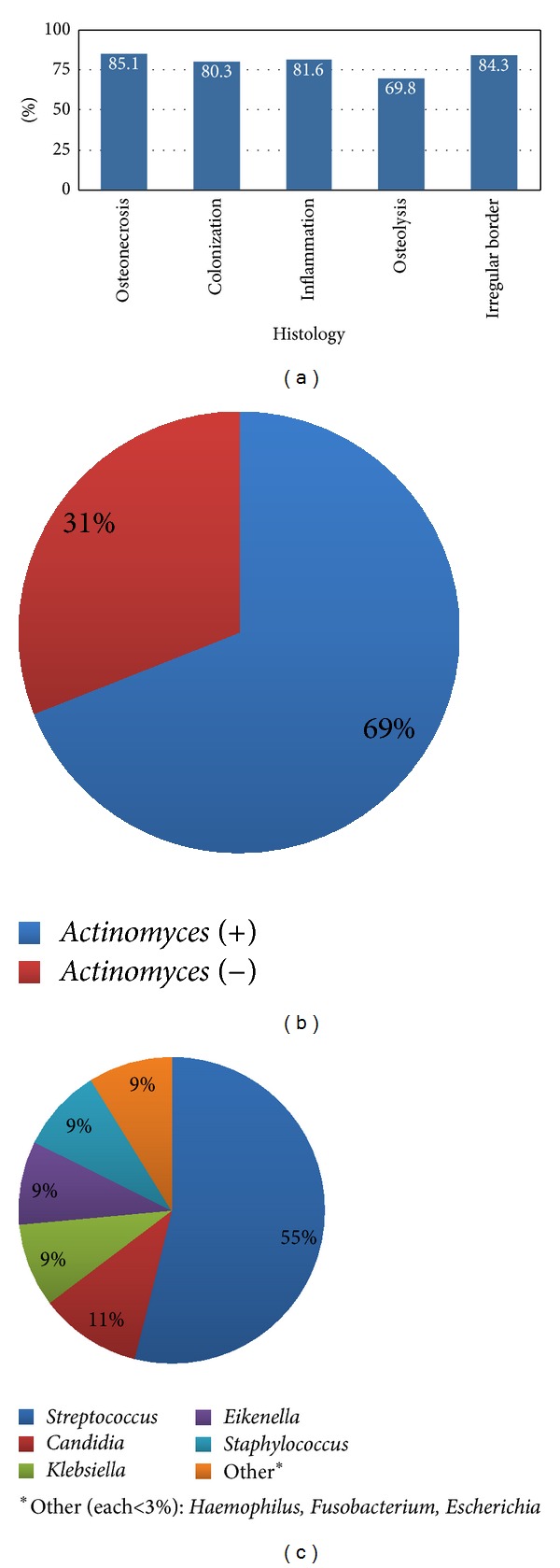
Histological results (a), microscopic identification of* Actinomyces* by H&E, Gram, and/or PAS stains (b), and culture results excluding* Actinomyces* (c).

593 (91.4%) had at least some level of histological data (Figure 3(a)). Necrotic bone was present in 375 (85.1%) samples along with inflammatory infiltrate and bacterial colonization in 270 (81.6%) and 172 (80.3%) cases, respectively. Nineteen (69.8%) reported osteolysis or “moth eaten” appearance and another 17 (84.3%) described irregular or “scalloped” borders.

Microscopic identification of* Actinomyces* occurred in 248 (68.8%) cases (Figure 3(b)). 166 cases obtained cultures with only 96 (57.8%) reporting growth (60.48%) or no growth (39.6%).

Excluding* Actinomyces*,* Streptococcus* was the most common organism grown from 19 (54.7%) reported lesions (Figure 3(c)). Other colonies grown (albeit much less frequently) included* Candida *(4),* Staphylococcus *(3),* Klebsiella *(3),* Eikenella *(3),* Haemophilus *(1),* Fusobacterium *(1), and* Escherichia *(1). Mixed oral flora (not otherwise specified) was reported in another 43 cases.

### 3.3. Treatment and Outcome

350 (60.7%) of cases received antibiotic treatment (83.0% PO, duration $\overset{-}{x}=8.7$ w, range 1.5–24 w; 16.9% IV, duration $\overset{-}{x}=2.9$ w, and range 1–6 w). Several articles mentioned antibiotic administration but were unfortunately nonspecific as to the route (191 cases; duration $\overset{-}{x}=6.3$ w, range 1–24 w). Unfortunately, antimicrobial rinses while being frequently reported were rarely specified to a specific patient/cohort and could not be quantified. 240 (66.8%) cases were treated with conservative surgery (i.e., superficial debridement, removal of bony sequestrum) involving local anesthetic and another 90 (22.3%) were treated with more radical measures (i.e., deep debridement, resection, etc.) under general anesthetic.

Complete recovery, partial recovery, and no recovery were reported in 108 (73.5%), 85 (47.0%), and 67 (24.7%) patients, respectively. Positive outcome NOS was reported in another 59 (53.4%) of patients. Time to described recovery in all cases showed $\overset{-}{x}=4.4$ w (range 1–52 w).

## 4. Discussion

To our knowledge, this is the largest and most complete retrospective analysis of BRONJ at the histological/microbiological level. The aim of this paper is to (1) educate the clinician of former/current diagnostic and treatment practices, (2) summarize histological and culture results, and (3) present this information within the context of a decade of BRONJ awareness and research. This work is limited by infrequent histological and microbiological reporting from case reports/case series, which have historically made up a significant portion of BRONJ literature. Possible overreporting secondary to selection bias of the results cannot be ruled out. Finally, a significant majority of the data stems from articles where it was not possible to assign a histological sample/culture to a particular patient. Thus, direct comparisons across datasets were not performed.

### 4.1. A Multifactorial Disease

Favia et al. observed with scanning laser microscopy that bone exposed to N-BPs shows minimal osteoclastic activity followed by deposition of newly formed, thicker bone with a diminished vascular supply [13]. A mosaic pattern of bone remodeling appeared strikingly similar to specimens from Paget's disease (another patient population prone to developing osteomyelitis) [14]. They suggested that the N-BP induced remodeling leaves cavities of bone isolated from marrow resulting in both necrosis and subsequent infection from colonizing bacteria. The present report overwhelmingly supports the presence of infection (81.6% with inflammatory infiltrate and 80.3% with bacterial colonization) in the setting of osteonecrosis (85.1%). However, these findings do not exclude the possibility that concurrent colonization and/or infection may be present during and even facilitate N-BP remodeling of bone.

### 4.2. *Actinomyces*, Oral Flora, and Biofilms

The filamentous, anaerobe* Actinomyces* has long been associated with the necrotic bone found in BRONJ lesions, but the exact role of the bacteria is unclear [15]. Colonization has been reported in varying frequencies ranging from as few as 39.0% to as high as 100% [9, 10]. Within this range, 248 (68.8%) of samples showed microscopic evidence of* Actinomyces* colonization and/or infection. The wide range observed in the literature may be explained by (1) the stage of disease in which a tissue sample was analyzed, (2) variation in criteria for determining the presence of* Actinomyces* (i.e., H&E, PAS, Gram stains, and/or necessary presence of tissue reaction), and (3) the stage of disease in which a tissue sample was analyzed [16]. Wei et al. showed with rRNA gene based sequencing considerably less* Actinomyces* colonization than reported with traditional methods [12]. This discrepancy is likely explained by the ease in which the filamentous* Actinomyces* is observed on microscopic analysis compared to other oral flora resulting in relatively higher qualitative reporting versus gene based techniques with the capacity to detect larger numbers of microorganisms both qualitatively and quantitatively.

While interesting, the clinical utility of knowing that* Actinomyces* colonization is abundant in BRONJ lesions has thus far been limited [17]. However, a retrospective analysis by Kaplan et al. regarding antibiotic treatment and* Actinomyces* bacterial load (number of colonies/surface area of tissue) showed a direct correlation between histomorphometric parameters of* Actinomyces* colonies and clinical course [18, 19]. The implications of a histological marker that correlates with clinical disease in the BRONJ patient population are exciting and may even have utility in other areas of BRONJ research.

Ganguli et al. showed that hydroxyapatite (HA) coated with the N-BP pamidronate was 60-fold more susceptible to bacterial colonization than HA alone [20]. Kos et al. postulated that it may be the NH_3_ (+) group of pamidronate acting as a steric factor to facilitate anchoring to the HA [21]. Further, they suggested that the ionic nature may even attract bacteria by direct electrostatic interaction (providing a mechanism for increased pathogenicity). Thus, in addition to bone remodeling, N-BPs may facilitate and select for growth of particular microflora. In our report, less than ten phylotypes were specified on culture (with a great majority of studies simply reporting mixed oral flora NOS) making it difficult to appreciate the biodiversity from culture alone. Culture-independent bioprofiling techniques emphasize the vast number of microorganisms and, more importantly, have shown that the BRONJ phylotype is significantly different than that seen in control groups [12].

It is unclear whether organisms present on culture or observed histologically (even if different compared to controls) are involved in the pathogenicity of the disease or are just simply present. Recent basic science, as well as clinical experience, suggests that the latter is increasingly becoming less likely. Tsurushima et al. histologically examined osteonecrotic lesions from Wistar rats (previously exposed to zoledronic acid) and observed significantly larger areas of necrotic bone in those specimens also inoculated with* Aggregatibacter actinomycetemcomitans *compared to normal saline controls [15]. This would suggest that certain phylotypes dominant in periodontal disease and BRONJ lesions, at the very least, act synergistically with N-BPs exacerbating bone remodeling and disease progression. For instance, a high abundance of* Streptococcus* and other aciduric bacteria has been suggested as causative factors in bone necrosis (and may even enhance growth of other aciduric bacteria) [12].* Streptococcus* was the most common phylotype growing in 54.7% of reported cultures. This finding is consistent with what is seen in culture-independent techniques [12].

*Streptococcus* is not alone for known pathogenicity. Mawardi et al. observed in a mouse model that* Fusobacterium* (reported in 1 cultures from our review) can directly cause BRONJ lesions and delayed epithelial wound healing (which both resolved after administration of a broad spectrum antibiotic regime) [22].

The most recent data suggests that individual phylotypes, however, may not be as important in comparison to how the microflora interacts as a whole. Sedghizadeh et al. described for the first time the presence of microbial biofilms consisting mostly of bacteria of various species (with occasional yeast) that were embedded in the extracellular matrix in BRONJ lesions [23]. Further, the biofilms were not present in control bone tissue. The biofilms consisted of Gram-positive and Gram-negative organisms, aerobes, and anaerobes/facultative anaerobes (i.e., typical oral flora). Unfortunately, conventional histopathologic techniques have not been useful in characterizing biofilms. Further characterization of the complex interactions between the microflora at this level likely represents the next stage of research in BRONJ infection pathophysiology [24].

In some cases, Sedghizadeh et al. described coaggregation (i.e., direct cell-to-cell recognition) of genetically distinct cell types [23]. Of particular interest, it should be highlighted that coaggregation was observed between* Actinomyces* species and coccal forms. We suggest that this cell-to-cell recognition with* Streptococcus* (the most reported phylotype by culture and culture-independent analysis) is significant.* Actinomyces* cell-to-cell recognition may be an alternative mechanism to explain why Kaplan et al. were able to correlate histomorphometric parameters (i.e.,* Actinomyces* bacterial load with a clinical course) [19]. For instance, the 10-year retrospective analysis may have been treating an underlying predominantly* Streptococcus* infection (blunting the acidic effect known to cause and exacerbate osteonecrosis) while using* Actinomyces* as a marker for such responsiveness.

Further, it is unlikely that* Streptococcus* is unique in this respect. It is well known that actinomycosis is predominantly a polymicrobial infection, and it should not be surprising that flora sensitive to the same antibiotics would mirror trends in response to treatment. This is consistent with penicillin as the predominant antibiotic (60% of cases) used in the Kaplan study to treat infection. Future research should assess the potential of* Actinomyces* as a potentially easily identifiable and inexpensive biomarker for both the presence of biofilms (i.e.,* Actinomyces* colonies at the surface of a biofilm with cell-to-cell contact, recent leave from a biofilm following pulsed shock, etc.) and BRONJ disease burden.

### 4.3. Immunocompromising Risk Factors

Bisphosphonate exposure to prevent bone destruction in patients with neoplastic disease remains the strongest risk factor with a 2.7- to 4.2-fold increase in the likelihood of developing BRONJ [25]. In our study, parenteral administration of bisphosphonates occurred in 81.8% (*n* = 516) versus oral administration in 18.0% (*n* = 95) while neoplastic disease accounted for 81.8% (*n* = 392) of the patient population. Malignancy frequently requires immunosuppressed states including chemotherapy (63.7%, *n* = 245) and/or steroid exposure (52.3%; *n* = 133) at some point during the clinical course. Other known immunosuppressed states associated with BRONJ included smoking (30.8%; *n* = 32) and diabetes mellitus (27.9%; *n* = 38) [26]. The cumulative effect of the above associations is illustrated at the microbiological level by the observed growth of* Candida* (typically seen in oral flora only in immunosuppressed states) in more than 10% of cultures.

### 4.4. BRONJ Clinical Manifestations and Acute Infection

The most common clinical manifestations of BRONJ in our analysis were pain (82.7%) and erythema (83.6%) followed by bone exposure (70.9%), pus (64.5%), and other extraoral manifestations such as lymphadenopathy, swelling, and draining abscess (57.8%). Thus, several of the most common clinical findings in patients presenting with BRONJ are also the classic signs of acute infection. Microbial infection alone is a causative factor in chronic, undiagnosed craniofacial pain and these patients are frequently misdiagnosed with trigeminal neuralgia or atypical facial pain (leaving the underlying infection untreated) [27]. The literature suggests that conservative regimens (i.e., nonsurgical treatment of infection with antimicrobials) have been effective at decreasing pain associated with BRONJ in the majority of the patient population [28].

### 4.5. Treatment and Outcome

Treatment recommendations for BRONJ lesions depend on clinical stage of disease and expertise of the physician. AAOMS guidelines suggest that Stage I (25.7% of our data) need only antimicrobial rinses. In Stage II (57.6% of our data), penicillin is recommended as empirical coverage unless relevant allergy or culture results dictate otherwise. Refractory cases may benefit from combined coverage, long-term coverage, or IV antibiotic therapy.

More recent reports since the 2009 guideline update suggest that combined surgical intervention (removing necrotic bone) along with antimicrobial rinses and empiric systemic antibiotic coverage (treating infected, viable bone) has been linked to complete healing in 70–87% of patients with Stages I and II of the disease [29]. A similar 73.5% (predominantly Stage II of the disease) showed complete recovery after a wide range of treatments in our report with results typically reported within a month after initiating treatment. Patients presenting in Stage III (16.7% of our data) will likely benefit from surgical debridement in combination with some form of antibiotic therapy. Deep debridement, resection, or other major surgical interventions were performed in 90 cases (22.3%).

## 5. Conclusions

After systematic review of the histological and microbiological data, the infectious etiology associated in BRONJ lesions should not be ignored. The authors recommend obtaining H&E, PAS, and Gram stain (all typically positive in the presence of* Actinomyces* colonies) along with the requirement of tissue reaction (i.e., inflammatory response or fibrosis) in the immediate vicinity to differentiate colonization versus infection when BRONJ biopsy tissue is obtained and/or reported. Refractory cases nonresponsive to antibiotics may benefit from an antifungal medication. Future research should examine the role of* Actinomyces* bacterial load as a potential BRONJ biomarker for disease burden, clinical course, and presence of biofilms.
